# Clinical Holistic Medicine: Teaching Orgasm for Females with Chronic Anorgasmia using the Betty Dodson Method

**DOI:** 10.1100/tsw.2008.116

**Published:** 2008-09-21

**Authors:** Pia Struck, Søren Ventegodt

**Affiliations:** ^1^ European Orgasm Academy, Copenhagen K, Denmark; ^2^ Quality of Life Research Center, Classensgade 11C, 1 sal, DK-2100 Copenhagen O, Denmark; ^3^ Research Clinic for Holistic Medicine, Copenhagen, Denmark; ^4^ Nordic School of Holistic Medicine, Copenhagen, Denmark; ^5^ Scandinavian Foundation for Holistic Medicine, Sandvika, Norway; ^6^ Interuniversity College, Graz, Austria

**Keywords:** Betty Dodson, orgasm, anorgasmia, desire, clinical holistic medicine, holistic manual sexology, sexological examination, adverse effects, side effects, masturbation, direct sexual stimulation, sexual and existential healing, therapeutic touch, clitoral vibrator, transference, ethics

## Abstract

The objective of this study was to test the Betty Dodson method of breaking the female orgasm barrier in chronic anorgasmic women. The aim was sexual and existential healing (salutogenesis) through direct confrontation and integration of both the repressed shame, guilt, and other negative feelings associated with body, genitals, and sexuality, and the repressed sexual pleasure and desire. We conducted a retrospective analysis of clinic data from holistic sexological manual therapeutic intervention, an intensive subtype of clinical holistic medicine (CHM). The patients received 3 × 5 h of group therapy, integrating short-term psychodynamic psychotherapy (STPP) and complementary medicine (CAM bodywork, manual sexology similar to the “sexological examination”). The therapy used the advanced tools of reparenting, genital acceptance, acceptance through touch, and direct sexual clitoral stimulation. A clitoral vibrator was used. Participants were 500 female patients between 18 and 88 years of age (mean of 35 years) with chronic anorgasmia (for 12 years on average) who were participating in the “orgasm course for anorgasmic women”; 25% of the patients had never experienced an orgasm. Our results show that 465 patients (93%) had an orgasm during therapy, witnessed by the therapist, and 35 patients (7%) did not. Postmenopausal women were as able to achieve orgasm as fertile women, as were women who never had an orgasm. No patients had detectable negative side effects or adverse effects. NNT: 1.04 < NNT < 1.12, NNH > 500. Therapeutic value: TV = NNH/NNT > 446. Our conclusions are that holistic sexological manual therapy may be rational, safe, ethical, and efficient.

## INTRODUCTION

The female orgasm is a variable, transient peak sensation of intense pleasure, creating an altered state of consciousness, usually with an initiation accompanied by involuntary, rhythmic contractions of the pelvic striated circumvaginal musculature, often with concomitant uterine and anal contractions, that resolves the sexually induced vasocongestion and myotonia, generally with an induction of well-being and contentment[1]. Findings from surveys and clinical reports suggest that orgasm problems are the first or second most frequently reported sexual problems in women[1,2,3,4]. Between 11 and 60% of adult women suffer from lack of orgasm[2,3,4], depending on factors such as culture and religion. On an individual level, self-insight and a positive attitude towards one's own genitals and sexuality are important.

Female anorgasmia is a significant sexual problem. The woman who lacks orgasm often also lacks desire and joy of sexuality, has low self-esteem, often feels like a sexual failure, and feels sexually wrong and ashamed of herself for not being the "woman she was meant to be". Perceptions of not being fully able to satisfy her partner sexually are normal and quality of life is often low[3]. The problem of female anorgasmia, from a psychodynamic perspective, often goes back to the parental lack of acceptance of the patient's genitals, body, and sexuality, often leading to intense feelings of shame and guilt, which seem to be repressed by a denial of physical and sexual needs, and accumulate in the pelvic and genital area[5,6]. Sexual abuse and sexual traumas from rape and incest often cause lack of orgasm[7,8,9,10]. If self-esteem is low, it is our clinical observation that there can be lack of orgasm from the simple psychological reason that the patient feels she does not deserve such pleasure, or does not know how to get it. It is very likely that anorgasmia is a socially inherited sexual dysfunction, but this has never been investigated scientifically. It is generally believed that anorgasmia, as most other sexual dysfunctions, is caused by a disturbed psychosexual development.

Holistic medicine has cared about female sexual problems from its very beginning[12], using, among other methods, the famous method of Hippocratic pelvic massage, often called "vaginal acupressure" or "vaginal massage" in the Nordic countries[13,14].

Holistic sexological manual therapy is a new and developing field, integrating efficient methods from standard medical sexology, Hippocratic medicine, and CAM (complementary and alternative medicine). There are many ways to work manually with female sexual dysfunctions in the sexological clinic; from simple therapeutic touch and acceptance through touch[6,15], to vaginal acupressure[12,13,14], and manipulation and stretching the pelvic muscles through the vagina. Direct sexual stimulation in the holistic medical clinic, i.e., with a clitoral vibrator, is a new, radical, and efficient approach, where the barrier created by accumulated and repressed shame, guilt, and lack of acceptance is taken down by direct and confrontational sexological work, taking the woman all the way to orgasm in the clinical setting[16]. Whenever there is a physical contact with the female genitals, there is a possibility for decharging emotions repressed to the tissues that can be used therapeutically[17].

The method can also be used for treatment of genital and pelvic pain[6,18,19,20], but we have not collected the data to document this in the present study. We believe the method used in this study helped the patients to heal not only sexually, but also existentially[21]. The group setting and the therapist's unusual willingness to use herself and her own sexuality as a tool in the therapy seemingly accelerated the process of healing[22] and caused no adverse effects. The many qualitative interviews with the patients indicated that the intervention also often alleviated mental, social, existential, and other problems[23,24,25,26,27], but this has not been quantitatively documented in this study.

## METHODS

The clinical holistic therapy used short-term psychodynamic psychotherapy (STPP) in combination with the advanced holistic tools of reparenting, genital acceptance, acceptance through touch, and direct sexual clitoral stimulation. The aim was sexual and existential healing (salutogenesis) through direct confrontation and integration of both the repressed shame, guilt, and other negative feelings associated with body, genitals, and sexuality, and the repressed sexual pleasure and desire. The CAM bodywork included the use of a clitoral vibrator.

When discussing the CAM bodywork use of clitoral vibrators, it must be presented in a manner that communicates the scientific nature of the process. In the U.S., the use of vibrators in funded research might be considered to be unethical or even illegal in some states. We understand that it might be difficult to get this presented research accepted in these states, however, Betty Dodson, Ph.D. did this work from 1973 to 1995 in New York City, and her method is now being used and further developed by many complementary therapists in the U.S. and Europe. We therefore find that it is time to address this kind of work scientifically, and we hope that we will be able to present it carefully so that this quite radical work in holistic sexology can be understood and appreciated.

The clitoral vibrator chosen for the treatment was a Hitachi Magic Wand, which has a very large head designed for efficient transference of vibrations to the vulva and clitoral region without causing soreness or irritation of the tissue. It can be used for an extended length of time compared to other vibrators, which is important when treating anorgasmic women with delayed orgasmic response. From a psychodynamic perspective, we find it interesting to note that bodily pleasure seems to be even more strongly repressed than emotional pain, and many layers of shame and unpleasant feelings must often be confronted before the female patient reaches the plateau of orgasm. This process needs prolonged sexual stimulation combined with therapeutic processing of emerging negative feelings and emotions, which is made possible by the specific design of this vibrator. Of course, this could also be done manually (compare the historical discussion below).

## The Therapeutic Intervention

We used the confrontational method developed by Betty Dodson. For 40 years, Dodson has been known in the U.S. as "the mother of female masturbation", and she has spent her life teaching women to accept their own bodies and sexuality, and to allow themselves the pleasure of genital satisfaction, alone and with a partner. Her motto is known from her many books and videos as "transforming masturbation to self-love". To our knowledge, this is the first time her method has been tested scientifically. The program has been adjusted to the Danish culture and changed according to our understanding of holistic therapy. Pia Struck developed a program of anatomical and physiological teachings, including Betty Dodson's films, followed by intensive existential group therapy with very strong elements of sexual confrontation, a concept that can be boiled down to "encountering and accepting your own body and sexuality, and your genitals in structure and functioning" as well as applying all of the means of the body in order to be able to encompass higher levels of sexual excitement. An important aspect of the therapy is the therapeutic energy work (level 7 in [16]), involving the patient's pelvic floor muscles and hip movements, making her push the pubic bone forward while tensing and releasing the musculature of the whole body, adding sound, complimented with different kinds of breath work, including holotropic breath work. Sexual fantasies are encouraged while she is stimulating all her erotic zones, including breast and nipple stimulation, and clitoral, vaginal, and anal stimulation. A key tool is thus the prolonged masturbation with variety and diversity. Every possible thing is done to make the female patient confront her emotional resistance related to her sexuality and to support her experience of a full body orgasm.

During therapy, the therapist will give the patient nurturing attention together with direct acceptance of digital contact of the vulva (level 8 in [16]). The sessions, in the nude, are started with a group psychodrama where every female patient takes on the role of a copulating male who is standing in the intercourse position of taking the female from behind. This allows for a deep emotional confrontation of the penetrating male energy and prepares the female patient to accept being the feminine pole. This is followed by a visual confrontation of their own genitals, in a room where everyone is nude, including the female therapist. In a later session, the clitoral vibrator (Hitachi Magic Wand) is used by everyone present in the room to induce orgasm. In this phase, the patient learns to engage her whole body in sexuality, and lets it free (freeing the Freudian "Id", or animal aspect of the human being).

Before the intervention (3 × 5 h of group therapy), written information on the intervention was given and before giving consent to the actual therapeutic procedure, a video introduction was given to the patients, demonstrating the procedures. After the course, the patients were instructed to masturbate according to a schedule for further improvement.

The patients were not told that they were participating in a study, as the project was planned to take place in two phases: a test phase not planned for publication, in which only the patients age, number of recent anorgasmic years, actual presence or lack of patient's ability to achieve orgasm on the course, and adverse effects, were registered, and a second phase now being planned, collecting much more detailed information. Because we have found the data already collected of significant scientific value, we have included them in our database for quality of life research and present them in the present paper. The protocol making the use of the data possible was approved by the local Scientific Ethical Committee of Copenhagen (see Acknowledgment). The combination of STPP and CAM led to holistic sexological manual therapy. The method used in this study is a highly confrontational style of CHM, where the therapy jumps right to the rehabilitation of genital sexuality; it is known from other similar studies that this kind of therapy is efficient in curing sexual and other health problems[28,29,30,31,32,33].

## The Therapist

The therapist, Pia Struck, is cochairman of the Danish Association for Sexology. Struck was trained in psychodynamic psychotherapy (1988–1991) and at the beginning of the study, she had 10 years of professional experience with the treatment of sexual dysfunctions, supplemented in 2001 with personal sexological training by Betty Dodson.

## Design and Setting

A retrospective analysis of clinic data from holistic sexological manual therapeutic intervention, an intensive subtype of CHM, was conducted. The patients received 3 × 5 h of group therapy, integrating STPP and complementary medicine (CAM bodywork, manual sexology similar to the "sexological examination" [see below]), and each paid 500 EURO for participation in the treatment program. Data were collected before and during visitation, and after the last session using interviews.

## Participants

Five hundred chronically anorgasmic female patients between 18 and 88 years of age (35 years on average) participated in the "orgasm course for anorgasmic women". On average, the patients had not had an orgasm for 12 years and one in four had never experienced an orgasm. Of these patients, 50 were treated individually (one-on-one) because they felt uncomfortable participating in the group.

The visitation procedure ensures that the therapist knows when participants have histories of childhood sexual abuse and ensures that they actually engage in a search for healing, not in an activity that allows them to recreate their past histories of abuse. Of the participants, 17% claimed that they had been sexually abused in childhood; eight patients reported that they had been diagnosed as being mentally ill, six with major depression, and these six patients all reported a significant improvement following therapy. Three patients dropped out during treatment. The patients that dropped out had no adverse effects from the therapy, such as reactive psychosis or retraumatization, and no serious events, such as mental hospitalization or suicide attempts. The patients were interviewed for about 1 h after the therapy, to ensure that the patients showed no signs of psychosis and no significant side effects from the course at this time. No patients reported side effects or experiences likely to be caused by retraumatization. There were no follow-up procedures, but everyone was encouraged to return for a free session if they had problems from the therapy later. No patients used this offer.

### Main Outcome Measures

Subjective experience of having an orgasm in combination with therapist's direct observation of the patient having an orgasm judged from "objective orgasmic behavior": impression of altered state of consciousness, involuntary rhythmic contractions of the pelvic and other musculature, vaginal and anal contractions, in combination with induction of well-being and contentment. The therapist visually monitored the vaginal and anal contractions. Every patient was interviewed qualitatively at the end of therapy to assess whether or not they had experienced orgasm, and the patient's experience was compared to the therapist's objective observations. Based on these data, it was concluded if the therapy had been successful in this regard or not.

## RESULTS

In over 112 courses held during 2001–2007 in Copenhagen and Aarhus, with three to six participants in each period and 500 patients in total, only one course in three had a patient that did not achieve an orgasm (a total of 35 patients, or 7%). Of the patients, 93% had an orgasm during the course that was witnessed by the therapist. Fifty patients needed individual therapy instead of group therapy. This was done successfully and there were no problems with transference and countertransference even though the female therapist and the patient were alone in the session. Problems arising from sexual transference and countertransference[34,35,36,37] can be hard to identify as such by the therapist, but are normally quite obvious for a supervisor[38]. The therapist was under supervision during the whole period of treatment, but the supervisor did not at any time notice any problems related to sexual transference or countertransference in the therapy.

Some of the 35 patients that did not obtain orgasm reported that they had an orgasm after the course, but this was not systematically registered because of limited resources for research and no follow-up procedure in this study. Many positive effects of therapy were reported, such as marked increase in self-esteem and quality of life, but these effects were not systematically investigated.

Postmenopausal women were as able to achieve an orgasm as fertile women, as were woman who never had an orgasm, but they became sore more often from using a vibrator. The results indicate that the etiology of orgasmic dysfunction is a disturbance of the female psychosexual development.

NNT (number needed to treat): As 93% of 500 patients (95% CI: 89–96%) were cured from anorgasmia, then NNT (number needed to treat to benefit) is calculated to be $1.04 < NNT < 1.12$.

### Adverse and Side Effects

In this study, all participants were specifically interviewed about any negative side effects of the treatment, and we very carefully evaluated if there had been any signs of sexual violation (i.e., signs of retraumatization) or complaints over experienced sexual violation (which most often is caused by transferences[34,35,36,37], but still must be thoroughly investigated), and we found none. Brief reactive psychoses and retraumatization were specifically looked for, but were not found.

Being able to identify and address adverse reactions should include a process carefully documented with objective criteria that protects the researcher and the participant. In this CAM study, this was simple. There were no reports at all of significant negative somatic or mental side effects, and thus no need of any objective evaluation of the side effects; the only adverse effect we found was soreness from the physical stimulation of the vulva, which was always temporary.

Patients who were severely sexually traumatized earlier in life often had some emotional difficulties, while their "old wounds" healed in the weeks after the therapy. Patients who were mentally ill (with diagnosed borderline conditions or major depression, sometimes on antidepressants) were included in the study and all reported feeling mentally better after the intervention. No patients had severe developmental crises or developed psychiatric disturbances, such as depression or psychosis.

Many patients reported positive additional benefits, such as increased desire and quality of life, higher self-esteem, better relationship with partner, etc. (see case reports at www.orgasmacademy.eu). The method of direct sexual stimulation in manual sexological therapy has no significant negative side effects or adverse effects.

NNH (number needed to treat to harm): As none of the 500 patients reported significant side effects, in spite of extremely confrontational sexological therapy, it is safe to conclude that even the most intensive and provocative tools of the advanced toolbox of CHM (STPP combined with CAM bodywork) are safe for the patients, if used correctly. NNH > 500. If we include the 500 patients treated in other studies with CHM, we find that NNH > 1,000[28,29,30,31,32].

Calculation of "therapeutic value" TV = NNH/NNT: Using the largest value of NNT, we find TV = NNH/NNT > 500/1.12 = 446.

## ETHICAL ASPECTS

The most important ethical safeguards that are in place to protect the participants and therapist are the following:

1. Full and complete information, including video demonstration of the content of the therapy.
2. Time to reflect about participation from video demonstration and the practical work.
3. Everything is done under supervision; the participants supervise every process in the group, and the supervisor carefully supervises the therapist. Supervision is done especially carefully when it comes to one-on-one therapy, and the therapist is not naked in these sessions. One-on-one therapy is only done because the patients do not want to be treated with other people in the room, as described above.
4. The therapy follows the ethical guidelines of the International Society for Holistic Health (ISHH) for holistic practitioners.
5. Careful follow-up, with interviews about adverse effects and therapeutic outcome (qualitative assessment) and evaluation of the therapy, is conducted.
6. The research team evaluates the process that is in place in the treatment organization to ensure that the treatment was done according the described methods and ISHH ethical standards.

Manual sexological therapy with direct sexual stimulation must be performed according to the highest ethical standards. The holistic sexological procedures are derived from holistic existential therapy, which involves reparenting, massage and bodywork, conversational therapy, philosophical training, healing of existence during spontaneous regression to painful life events (gestalts), and close intimacy without any sexual involvement[13,14]. In psychology, psychiatry, and existential psychotherapy, touch is often allowed, but a sufficient distance must always be kept between the therapist and client, all clothes kept on, and it is even recommended that the first name is not used in order to keep the relationship as formal and correct as possible. The reason for this distance is to create a safety zone that removes the danger of psychotherapy leading to sexual involvement.

The female patients in holistic existential therapy and holistic sexology with life-long anorgasmia often find their situation pretty hopeless; many of them have been dysfunctional and incurable for many years or they suffer from conditions for which there has been no efficient biomedical or psychotherapeutical cure. They suffer from a condition that is a serious burden to their marital life, if they have a husband; often the problem makes them unable to find or keep a partner. Often the problem of anorgasmia is caused by traumas from earlier sexual abuse, which needs more effective and direct tools for the induction of healing (salutogenesis).

The primary purpose of holistic existential therapy is to improve quality of life, secondarily to improve health and ability. The severe conditions of the patients and the chronicity are what ethically justify the much more direct, intimate, and intense method of holistic manual sexological therapy, which integrates many different therapeutic elements and works on many levels of the patient's body, mind, existence, and personality at the same time. Holistic sexology is holistic existential therapy taken into the domain of sexology. The general ethical rule is that everything that does not harm and in the end will help the patient is allowed ("first, do no harm"), but we understand that this procedure is not accepted in many other countries due to sexual taboos and legal regulations.

It is interesting though that this, or similar techniques, has been used for centuries by many physicians[13,14] and, in particular, alternative therapists outside the medical profession[13,14]. An important aspect of the therapy is that the physician must be creative and in practice invent a new treatment for every patient. To use a sexological technique involving direct sexual clitoral stimulation, the holistic sexologist must be able to control his/her behavior and must strictly avoid the danger of the therapeutic session turning into mutual sexual activity. The necessary level of mastery of this art can only be obtained through training, supervision, and preferably a third person present, which is one good reason for doing this in a group setting. Sometimes the patients are too shy to have more than the therapist present in the room; in this case the therapy can only be done one-on-one and this has not caused problems. The role of the sexologist is parentally accepting, generous and supporting, loving and therapeutic.

The treatment included patient masturbation under supervision and instruction. The client uses, under the therapist's instruction, the clitoral vibrator after initial digital stimulation.

In this study, 500 female patients with often lifelong anorgasmia received direct sexual stimulation of the clitoris during the therapy and 93% of the patients experienced that the procedure solved their problem. The success of this study gives us one more very important tool for holistic medicine. Together with the other tools of holistic manual therapy, such as acceptance through touch and acupressure through the vagina, we now have tools for solving problems related to female sexual dysfunction. We therefore conclude that direct sexual stimulation can be a safe and efficient procedure, and an important new tool in the holistic medical toolbox.

## DISCUSSION

### The History of Direct Sexual Stimulation as Sexological Therapy

Direct sexual stimulation of women has a long history in medicine. Before use in the sexological examination (see below), it was used in holistic medicine for millennia. In the Corpus Hippocratic, "hysteria" is described as a disease caused by the energies related to the womb, treatable with exercise and pelvic massage[12,39]. In the first century AD, Celcus and Soranus recommended genital massage for hysteria[40,41]. Aretaeus Cappadox recommended the same in the third century AD based on Hippocrates[42]. Galen (ca. AD 129–200) also understood "hysteria" to be caused by lack of psychosexual development[43]. He carefully describes, obviously based on Hippocrates, the procedure of genital massage therapy, resulting in the contractions and the release of fluid from the vagina, after which the patient was relieved of her symptoms. (The great respect for Hippocrates and Galen made the procedure of genital massage a standard procedure until the end of the 19th century[44].) In "Tetrabiblion", Äetius of Amida (502–575) described a uterine contraction, muscle spasm of the entire body, and discharge of fluid from the vagina[45]. Muschion's *Gynaecia* describes the procedure a little differently as manual therapy of the vulva[46]. Rhazes, an Arab physician, described a similar procedure around 900 AD[44, p. 24]. It is not clear from most medieval sources whether the manipulation of the female genitals needed to go all the way to orgasm to be therapeutic[43], but many sources write that it is necessary to continue the massage treatment until the vagina discharges its fluid, indicating that the physicians massage must be sexually stimulating for the therapy to work[43, p. 22ff], and the descriptions of universal muscle contractions mentioned above leave little doubt that the treatment was taken all the way to orgasm. It is clear that many different styles of genital massage have been used, some concentrating on raising the energy of the uterus, such as Hippocrates, and others, such as Muschion and Rhazes, focusing more on sexual stimulation of the vulva. The many historical sources leave little doubt that physicians have successfully used this kind of massage therapy for millennia to heal the female patient and free her from severe physical, mental, existential, and sexual problems. The rationale for such a therapy that seems strange and quite mysterious for most modern physicians is that there is a close statistical connection between sexual maturity and physical and mental health and general well-being, as recent research has documented[47,48,49,50,51,52,53,54,55,56,57,58,59,60,61].

## Comparison with the Traditional Sexological Examination

In the middle of the last century, Reich, Hartman, Fithian, Morgan, Hoch and other researchers in sexology developed a sexological intervention they called "sexological examination"[62,63,64,65,66, 67,68,69]. With regard to the obvious above-mentioned ethical problems of treatment with direct sexual stimulation, we must emphasize that this "sexological examination", from the beginning, included the method of direct sexual stimulation[66]. The method is in contemporary use by Hartman, Fithian, and many others. Hartman and Fithian noted that they do not, as a part of the examination, intentionally stimulate the patient to "a high level of arousal", but "some women do become aroused, and occasionally a sex flush will be observed in the process practice of the vaginal caresses"[69].

The sexological examination involves all parts of the genitals, including the vagina, the labia minores and majores, and the clitoris[63,64,65,66,67,68,69]. It is noteworthy that the tradition of sexological examination seemingly has been without ethical difficulties; the reason for this is presumably that everything in the sexological clinic happens after consent and obviously is justified by the severity of problems of sexual dysfunction that often completely destroy the patient's sexual and marital life.

Some of the obligatory steps of the sexological examination are, according to Hartman and Fithian[69]:

- Acquainting the female with her own body to dispel some of the feeling that the genital area is a special place forbidden for all but physicians to see.
- Searching for areas where nerve endings come together in a systematic way, suggesting that this may develop positive feelings.
- Assisting women in determining areas of perception, feeling, and awareness in their vagina. Pointing out areas in the vagina that tend to be more sensitive and responsive for many women (i.e., 12, 4, and 8 o'clock positions).
- Determining a woman's response and arousal patterns. Indicating to her whether or not she lubricates well and vasocongests when she does.
- Locating areas digitally that may be producing pain, discomfort, or problems with sexual arousal or intercourse — such as separation of muscle in the vaginal wall; long labia minora; scarring, which may be tender or fibrous — and to pinpoint the source of "pain" when present.
- Determining whether or not clitoral adhesions are present is more important than the stimulation of the clitoris in the female sexological examination. This is a condition where the prepuce is stuck or adhered to the glans clitoris. For preorgasmic women, the inability of the clitoris to withdraw as part of sexual arousal may prevent particular women from full response. Even though some women are orgasmic with clitoral adhesions, freeing them usually results in easier, quicker orgasms and less discomfort due to calcified, trapped smegma.

- Identifying, where present, reasons for vaginismus, which are not only physiological, but psychological.

The sexological examination was called an examination, not a treatment, presumably to make it more acceptable to the public, but it has always been as much a treatment as an examination[62,63,64,65, 66,67,68,69]. When we consider this, we must conclude that the method of direct sexual stimulation in itself is not remarkable or problematic; it is a traditional sexological tool for treating sexual dysfunction. What is different with the Betty Dodson method is the obligatory step of supporting the patient in going all the way to experiencing a full orgasm during the therapy.

## Discussion of Holistic Manual Sexology

The holistic manual sexological therapy used in this study was performed in the feministic tradition of nudity, expressive sexuality, genital self-exploration with mirrors, and common masturbation in a group of females. It also built on the 40-year-long process of sexual liberation in western societies, making it unproblematic for Danish women of all ages to participate in the group and share their sexual problems. The efficient elements of the highly confrontational method for breaking the female orgasm barrier in patients with anorgasmia seem to be reparenting, genital self-touch, acceptance through touch, and direct sexual clitoral stimulation in therapy, allowing all the difficult feelings associated with the genitals, pelvis, body, and sexuality to emerge in order to be processed in therapy. A clitoral vibrator was used.

In the traditional sexological examination, the therapist is not naked, the therapist is often male, and therapy happens without the common masturbation that seems to be an important aspect of Dodson’s treatment, presumably because of the emotional resistance provoked by this radical procedure. The patients are normally not taken all the way to orgasm, although this occasionally happens, without this becoming a problem or in conflict with the ethical rules. On the other hand, Betty Dodson sometimes avoids the digital stimulation of her female patients’ genitals that is obligatory in the sexological examination, by using the mechanical stimulator. The specific features of masturbation and nudity that makes Betty Dodson’s method spectacular and somewhat alienating, controversial, and strange to normal therapists, especially if they are trained as physicians or nurses, seem to be there to enhance the therapeutic resistance work. Other elements, such as direct sexual stimulation, are, in spite of their radical nature, traditional elements of the “sexological examination”, and also of the Hippocratic tradition of psychosexual healing and salutogenesis. All of this indicates that the tool of direct sexual stimulation of the patient should be accepted as a usable therapeutic tool, and added to the advanced holistic medical toolbox.

We know for sure that many therapists would not like to masturbate naked with their patients in the future. We also now know that direct sexual stimulation is one of the most powerful tools of holistic sexological manual therapy and has been so for millennia. As 11–60% of all women on the planet seems to be struggling with anorgasmia, and problems related to sexual pleasure and desire often destroy their sexual life or hinder them in having a happy and successful marital life, we cannot afford to be “tight” and moral in our attitude towards this powerful medical tool, and disappoint the patients that so desperately need it.

Manual sexological work that takes the female patient to orgasm creates a huge arousal in the patient and can also, obviously, be sexually gratifying for the therapist, also without the therapist acting out. Sexuality is pleasurable. This is how we are made as human beings and there is nothing we as sexually normal therapists can do about it. However, we can be extremely certain that we have the training and the ethics needed to prevent us from acting out sexually towards our patients during therapy. In this study, we very carefully evaluated if there were any signs of sexual violation or complaints of experienced violation

(which most often is caused by transferences, but still must be thoroughly investigated), and we found none.

The correct indication for direct sexual stimulation needs some reflection. The Hippocratic physicians used hysteria and poor psychosexual development as sufficient indication; in our study the indication was anorgasmia. As it is well known from recent research[47,48,49,50,51,52,53,54,55,56,57,58,59,60,61], psychosexual development and mental illness seem to be very closely related, as Hippocrates, Galen, Freud, Jung, Reich, and many more physicians have realized.

Direct sexual stimulation can obviously be used to facilitate the sexual development of the anorgasmic woman (often described as caught in infantile autoerotism). The next important research question is if the much more subtle shift from immature clitoral sexuality, closely correlated to neurotism and immature psychological defenses[47], to mature vaginal sexuality can also be facilitated by the tool of direct sexual stimulation. Our own findings here and in other studies[13,14,28,28,30,31,32,33,37], and the above-mentioned findings of Brody et al. of strong associations between genital maturity and a number of aspects of health[47,48,49,50,51,52,53,54,55,56,57,58,59,60,61], justifies such a study. From a theoretical perspective, it is highly likely that the resistance work done in this kind of therapy is quite unique and therefore of great value when other methods fails.

It is not clear to us if there are ethical problems connected with using the diagnosis of immature sexuality as an indication for treatment with direct sexual stimulation. Obviously, there have been no ethical problems with this treatment or any such indication throughout the history of European physicians, so it is hard to see why there should be ethical problems today, as long as the intent is to help the patient and as long as the treatment is documented to give this help.

We must remember that every procedure that allows the therapist to take the patient into her resistance will be therapeutic and helpful, so the wise answer might be to let the resistance of the patient guide the choice of the proper therapeutic tools of the holistic sexological treatment as recommended by Reich, the founder of sexology[62].

## CONCLUSIONS

Lack of orgasm is a very serious sexual problem for countless millions of women in our time. As 11–60% of all women seems to suffer from lack of orgasm, without getting the help they need from their physician, gynecologist, psychotherapist, or sexologist, they often live their whole lives with anorgasmia and related problems. Most surprisingly, almost all women can learn to have an orgasm in holistic therapy, with 93% of female patients experiencing orgasm after only 15 h of holistic sexological manual therapy using the tool of direct sexual stimulation[16], in spite of the patient never having had an orgasm. Postmenopausal woman are just as able to achieve orgasm as fertile women in the therapy.

The female orgasm barrier seems to be caused primarily by the parental lack of acceptance of the girl's genitals and sexuality, and other sexual traumas, leading to arrested psychosexual development. This theory seems to be in accordance with the presented results: this can be healed simply by giving acceptance to the body, sexuality, and genitals that the patients never received, and thereby rehabilitating the lost self-acceptance. A combination of parental acceptance, acceptance through touch, and direct sexual clitoral stimulation allowed the patients to confront and let go of the shame, guilt, and other negative feelings that had been connected to their genitals and sexuality during their upbringing. Holistic sexological manual therapy is extremely efficient, and sexologists who are trained in psychodynamic psychotherapy and ethics of therapy can use the tools of direct sexual clitoral stimulation when psychodynamic psychotherapy and less sexually confrontational bodywork cannot solve the female patients' problems related to orgasm and sexual desire.

Manual sexological examination and treatment has been a practical tool in sexology for at least 50 years, and before that it was a part of the holistic medical tradition. Holistic sexological manual therapy is therefore likely also to be efficient with other sexual dysfunctions, such as lack of sexual desire, vaginism and genital pain/vulvodynia, low sexual self-esteem, and poor psychosexual development in general, but this needs further scientific investigation.

To prevent the huge problem of female anorgasmia in future generations, we need to work on a societal and cultural level to develop parental positive attitudes towards the child's sexuality, body, and genitals. Of course, this will start with the parents learning to accept their own bodies and sexuality, especially the mothers learning to have orgasms themselves, as it judged from the present findings that anorgasmia most likely is a socially inherited sexual dysfunction.

## ACKNOWLEDGMENTS

The research in quality of life and complementary medicine (CAM/CHM) was approved by the Copenhagen Scientific Ethical Committee under the numbers (KF)V. 100.1762-90, (KF)V. 100.2123/91, (KF)V. 01-502/93, (KF)V. 01-026/97, (KF)V. 01-162/97, and (KF)V. 01-198/97. The study was funded by the IMK Almene Foundation. We declare no conflict of interest.
